# Effect of listeriolysin O (LLO) secreted by listeria monocytogenes on apoptosis of macrophages derived from patients with macrophage activation syndrome- in vitro

**DOI:** 10.1186/1546-0096-12-S1-P215

**Published:** 2014-09-17

**Authors:** Reza Shiari, Negin Kohkar, Abdollah Karimi, Sudabeh Taheri

**Affiliations:** 1 Pediatric Rheumatology, Shahid Beheshti University of Medical Sciences, Tehran, Iran, Islamic Republic Of; 2 PIRC, Shahid Beheshti University of Medical Sciences, Tehran, Iran, Islamic Republic Of; 3 Immunology, Shahid Beheshti University of Medical Sciences, Tehran, Iran, Islamic Republic Of

## Introduction

The term of macrophage activation syndrome (MAS) refers to a condition caused by excessive activation and expansion of T lymphocytes and macrophagic histiocytes that exhibit hemophagocytic activity. The expansion of these cells also leads to a massive systemic inflammatory response associated with pan cytopenia, liver dysfunction, and coagulopathy consistent by disseminated intravascular coagulation (DIC).

MAS has been reported in association with almost any rheumatic disease and most common in *systemic onset JIA.* However, the pathological mechanisms of MAS are not fully understood.

## Objectives

In clinically similar primary HLH, the uncontrolled proliferation of T cells and macrophages has been linked to decreased natural killer (NK) cell and cytotoxic T cell function, often due to mutations in the gene encoding perforin. Deficient cytotoxic function lead to Inefficient apoptosis and overactivated macrophages.

Because high doses of LLO are known to cause cell death by necrosis or apoptosis, we decided to evaluate the effect of LLO on apoptosis of macrophages derived from patients with macrophagea ctivation syndrome- In vitro.

## Methods

Blood from MAS patients and healthy donors was collected in Falcon tubes containing EDTA at 2 mM final concentration and incubated with enrichment antibody cocktail (50 µl per ml of whole blood) at room temperature for 20 minutes. Cells were then separated by density gradient using Ficoll-Paque™ PLUS (GE Healthcare). Platelets present in the enriched monocyte fraction were discarded by 3 washing steps in PBS, 2% FBS. Finally, monocytes were seeded in RPMI 10% FBS, 4 mM L-Glutamine with Pen/Strep at a concentration of 5×10^5^ cells/ml in 12-well tissue culture treated plates for 6 days. The effects of various concentration of LLO ( 10%, 25%, 50%, 75% and more) were evaluated on apoptosis of macrophages in both groups.

## Results

By using LLO less than 75%, there were no apoptosis in both normal and patients groups. However, 50% of macrophages of healthy donors and 82% of MAS patients showed apoptosis by LLO 75% and more.

## Conclusion

High concentrated LLO may induce significant apoptosis in macrophages derived from patients with Macrophage Activation syndrome- In vitro.

## Disclosure of interest

None declared.

